# Gene-Environment Interactions in Inflammatory Bowel Disease: A Systematic Review of Human Epidemiologic Studies

**DOI:** 10.1093/ecco-jcc/jjaf061

**Published:** 2025-06-04

**Authors:** Jingjing Bai, Dianne Gelien Bouwknegt, Rinse Karel Weersma, Gerard Dijkstra, Kimberley Wilhelmina Johanna van der Sloot, Eleonora Anna Margaretha Festen

**Affiliations:** Department of Gastroenterology and Hepatology, University of Groningen, University Medical Center Groningen, Groningen, the Netherlands; Department of Gastroenterology and Hepatology, University of Groningen, University Medical Center Groningen, Groningen, the Netherlands; Department of Gastroenterology and Hepatology, University of Groningen, University Medical Center Groningen, Groningen, the Netherlands; Department of Gastroenterology and Hepatology, University of Groningen, University Medical Center Groningen, Groningen, the Netherlands; Department of Gastroenterology and Hepatology, University of Groningen, University Medical Center Groningen, Groningen, the Netherlands; Department of Gastroenterology and Hepatology, University of Groningen, University Medical Center Groningen, Groningen, the Netherlands; Department of Genetics, University of Groningen, University Medical Center Groningen, Groningen, the Netherlands

**Keywords:** genetics, gene-environment interaction, inflammatory bowel disease

## Abstract

**Background and Aims:**

Complex gene-environment interaction (GXE) for inflammatory bowel disease (IBD) remains elusive. This systematic review aims to summarize the current evidence of GXE in IBD.

**Methods:**

PubMed, EMBASE, Web of Science, and Scopus were systematically searched from inception through April 30, 2024, to identify publications examining the interaction effect of genetic variants and environmental factors in IBD. All eligible studies were graded using STREGA guideline.

**Results:**

Four thousand eight hundred thirty-three publications were identified and screened, resulting in 39 eligible studies, and 17 studies reported statistically significant interactions. *NOD2*-smoking interaction was most frequently investigated and showed variant-specific effect at rs2066847 regarding the risk of Crohn’s disease. Gene-smoking interactions were further identified in other IBD risk genes (*ATG16L1*, *IL23R*, and *CALM3*), detoxification genes (*GSTP1* and *HMOX1*), smoking-associated genes (*CHRNA3*, *CHRNA5*, *PPP1R3C*, and *BDNF*), and the inflammatory cytokine (*IL1B*) through a candidate gene approach. Immunochip-wide interaction analyses yielded 64 smoking interacting variants. Gene-diet interactions were observed across multiple nutritional measures, including fatty acid intake with *CYP4F3* and *FADS2*, serum selenium with *SEPHS1* and *SEPSECS*, potassium intake with *IL21*, alcohol consumption with *IL12B*, heme iron intake with *FCGR2A*, and serum vitamin D with *VDR*.

**Conclusions:**

Current evidence indicated that the IBD risk conferred by environmental factors can vary among the individuals carrying certain genetic variants. Further efforts, including genome wide environment interaction studies and genotype-based nutrition/lifestyle clinical trials, are needed to unravel the missing heritability influenced by environmental exposures and to construct personalized recommendations of lifestyle/dietary modification based on an individual genetic background.

## 1. Introduction

Inflammatory bowel disease (IBD), consisting of Crohn’s disease (CD) and Ulcerative colitis (UC), is a group of chronic inflammatory disorders of the gastrointestinal tract, affecting more than 4.9 million people worldwide.^1^ The cause of IBD is known to be multifactorial with complicated interactions across genetics, environmental exposures, an aberrant immunological response, and microbiome.

Enormous progress has been achieved in genetic research for IBD. So far, 320 IBD-related loci have been identified by genetic association studies through collaborative efforts of the International IBD Genetics Consortium (IIBDGC).^2^ However, the disease variance explained by IBD-associated loci is still rather modest and differs across European (EUR) and East Asian (EAS) populations.^2^*TNFSF15* and *NOD2* demonstrated the most striking difference in explained disease variance between EAS and EUR with 5.5% and 1.9%, respectively, which is largely driven by the difference in allele frequencies (*NOD2*) and the effect size (*TNFSF15*) between EUR and EAS.^2^ The well-established CD causal variants in *NOD2* found among EUR showed substantially low composite allele frequency in EAS, while the magnitude of the *TNFSF15* effect is much higher in EAS than EUR.^3^ The heterogeneity of genetic contribution across different ancestries might be influenced by clinical heterogeneity, gene-gene interaction, and gene-environment interaction.^2,3^

In the context of genetic epidemiologic studies, gene-environment interaction was defined as “different effect of an environmental exposure on disease risk among the subjects with different genotypes’’ or “different effect of a genotype among individuals with different environmental exposures.”^4^ Gene-environment interaction has been widely investigated in etiological studies for chronic complex diseases such as breast cancer, type 2 diabetes, and psychiatric diseases to examine its role in disease risk and to identify subpopulations at highest risk when genetic variants and environmental risk factors are jointly present.^5–7^

Western countries are currently in the compounding prevalence stage with a relatively stable incidence and rapidly increasing prevalence of IBD, while the incidence of IBD in most newly industrialized regions is at the acceleration stage.^8^ The co-occurrence of modernization processes and rapid increase of IBD incidence in newly industrialized countries suggests a strong influence of environmental exposures such as westernized lifestyle, dietary patterns, and industrial pollutants, collectively referred to as exposome, on the development of IBD.^9^ Therefore, the pivotal role of the exposome regarding the risk of IBD is being investigated intensively. A considerable number of associated environmental factors are now emerging from observational studies focused on lifestyle and hygiene, surgeries, exposure to drugs, dietary intake, microorganisms, and vaccinations.^10^ However, due to the low consistency in results across different studies, few environmental factors demonstrated high credibility.^10^

These discrepancies might not only be attributed to the methodological differences or unstandardized definition of environmental factors but also, or even more importantly, the genetic architecture of the study populations. Previous evidence consistently showed that the distribution of allele frequencies of multiple xenobiotic metabolizing enzymes differs significantly between populations. For example, the functionally deficient *CYP2A6* alleles, involved in nicotine metabolism, are more prevalent in EAS than EUR.^11^ Regarding the etiopathogenesis of CD, the effect of dietary exposures on CD risk was postulated to be modified by an individual’s metabolizing capacity influenced by genetic variants in xenobiotic metabolizing enzymes.^12^ In addition, high intake of unsaturated fatty acids might be more damaging among individuals carrying coding variants in proinflammatory cytokine genes.^12^ These proposed hypotheses have led to an increasing amount of research articles exploring the different effects of specific environmental exposures on IBD risk among the individuals with different genotypes or vice versa.

Despite previous reviews emphasizing the importance of effect modification of environmental exposures in disease pathogenesis of IBD and highlighting the complexity and challenges of performing large-scale gene-environment interaction research, to date, no comprehensive overview of existing findings on this complex area of research is available.^13,14^ While the concept of interaction can be different under specific contexts, population-level epidemiologic GXE interactions are the main focus in our current study.^15,16^ Hence, we do not include those studies solely focusing on the effect of environmental factors/genetic variants and those investigating broader correlative relationship between environmental factors and genetic variants, such as mendelian randomization studies and host genetics-microbiome correlation studies. Such studies, though valuable, fall outside of our focus. By narrowing this scope, we aim, in this article, to systematically summarize and assess the quality of current evidence of gene-environment interaction studies in IBD, which may guide future pathophysiological studies and aid clinicians and policymakers in identifying patient subgroups that could gain maximum benefits from preventative interventions.

## 2. Methods

### 2.1. Search strategy

A systematic searching was performed in four literature databases (PubMed, EMBASE, Web of Science, and Scopus) from the inception through April 30, 2024, to identify research articles examining the interaction effect of genetic variants and environmental factors in IBD. We used a combination of MeSH terms and text words covering both genetic and previously reported environmental factors in IBD and limited the search to the English language (Supplementary Methods). The reference lists of potentially relevant papers and previous literature reviews were screened to comprehensively capture the relevant studies. The search results were first imported to EndNote for de-duplication.^17^ Subsequent independent screening was performed via systematic review web application Rayyan by 2 investigators (J.B. and D.B.).^18^ Disagreements were resolved by consensus discussion (J.B., D.B., E.F., and K.S.). The systematic review protocol was registered on PROSPERO (CRD42023443071).

### 2.2. Selection criteria

#### 2.2.1. Types of studies

The present review was restricted to human epidemiological studies irrespective of study designs. Observational studies, including case-control, case-only, and prospective cohort studies were all collected and summarized. The intervention studies focusing on genotype-based environmental/lifestyle or dietary interventions, such as physical activity, smoking, weight loss, lifestyle education, and dietetic consultation were included. Studies were excluded if they only performed experiments on human cells, tissues, or animal models without human epidemiological data. Unpublished articles, case reports, reviews, and conference abstracts were excluded.

#### 2.2.2. Types of participants

No restriction based on age, sex, ethnicity, study setting, or other characteristics of participants was applied.

#### 2.2.3. Types of genetic variants

Single nucleotide polymorphisms (SNPs), microsatellites, and any other kind of genetic variants (e.g. insertion, deletion, and copy number variations) were included. Studies incorporating IBD polygenic risk scores were also included.

#### 2.2.4. Types of environmental factors

Studies focused on IBD-associated environmental factors were included independent of the collection methods (eg, structured questionnaires, medical charts, or touch screen survey).

#### 2.2.5. Types of interaction analysis methods

Studies assessing multiplicative, additive interaction, or conducting strata association analysis of genetic variants at different levels of exposures or vice versa were included. Gene-gene interaction or gene-environment association/correlation studies were excluded. Mendelian randomization studies were not included.

#### 2.2.6. Types of outcomes

Risk of development of IBD, disease activity, and all types of IBD-related complications (eg, fistula, strictures, surgeries) were included.

### 2.3. Data extraction and quality assessment

The characteristics of eligible studies including first authors, year of publication, study design, sample size, genetic variants, implicated genes, environmental factors, statistical methods for interaction analysis, outcome(s) studied, and main findings were extracted by 2 investigators independently (J.B, and D.B.). We evaluated the study quality based on the Strengthening the Reporting of Genetic Association Studies (STREGA) checklist.^19^ The included studies were categorized into high, moderate-high, moderate-low, and low quality if the studies met more than 75%, 50%-74%, 25%-49%, and ≤24% of the criteria, respectively.

## 3. Results

**Figure 1.**
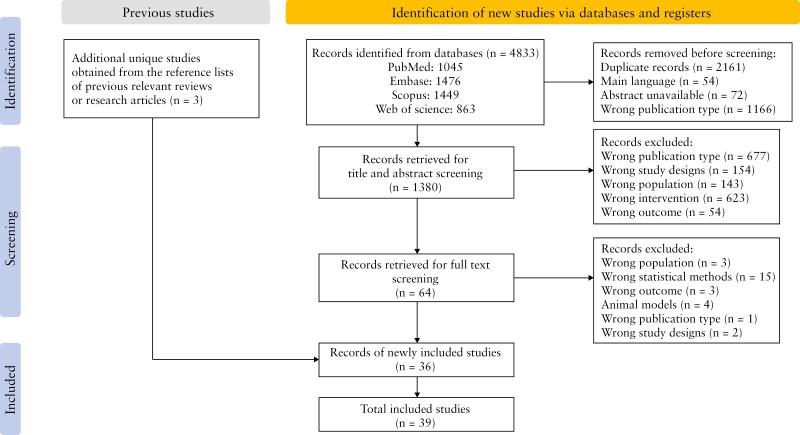
PRISMA flow diagram of study selection.

Four thousand eight hundred thirty-three literature studies were identified, of which 64 articles suited our research purpose. After full-text screening, 39 articles fulfilled the selection criteria, and 28 studies were excluded based on their studied outcome, statistical analysis, study designs, etc. (Supplementary Table). Finally, 3 additional articles were obtained from the reference list of previous reviews and potentially relevant articles (Figure 1).

### 3.1.1. Characteristics of included studies

**Figure 2.**
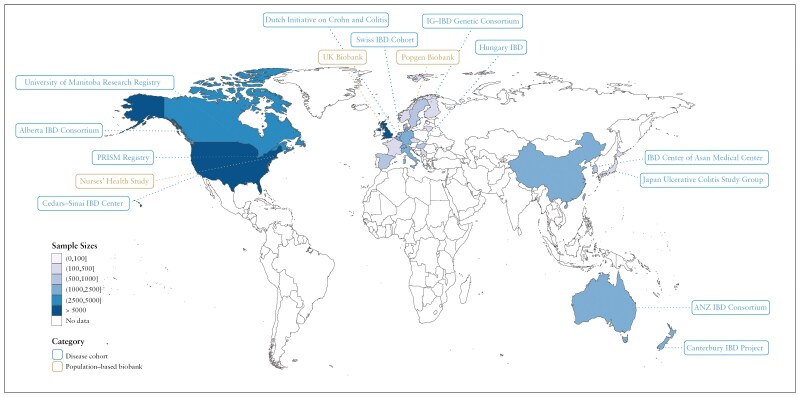
The global distribution of gene-environment interaction studies in IBD. ANZ IBD consortium, Australia New Zealand IBD Consortium. IG-IBD, Italian Group for the study of Inflammatory Bowel Disease. PRISM study, Prospective registry of patients with IBD at the Massachusetts General Hospital.

**Figure 3.**
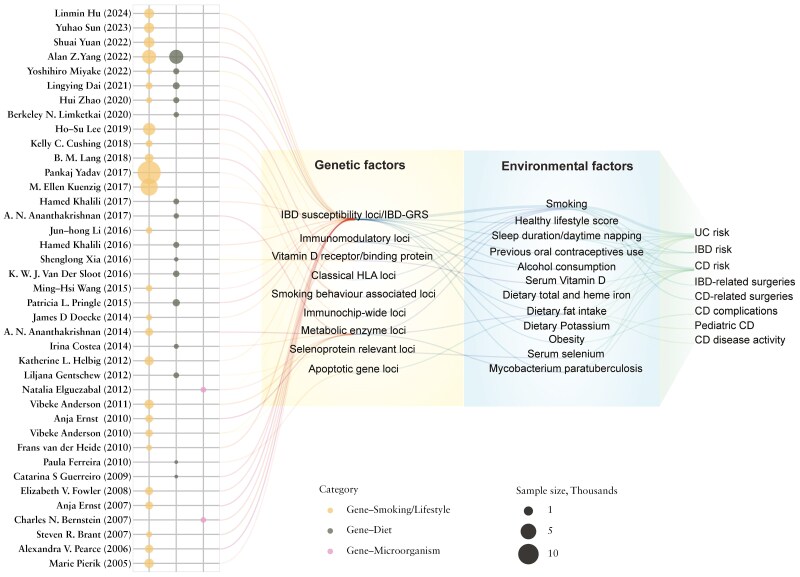
The overview of gene-environment interaction studies in IBD. The area of the bubbles reflected the sample size of IBD patients included in each study. The orange bubbles are the studies focusing on Gene-smoking/lifestyle interactions. Dark green ones stand for gene-diet interactions. Pink bubbles are for gene-microorganism interaction. Each line represented a gene-environment interaction examined in the individual study. Alan Z. Yang et al. (2022) explored the interactions of 38 candidate environmental exposures and IBD GRS for IBD occurrence. Due to the limited space, only significant interactions were displayed for this study. GRS, Genetic risk score.

A wide variation in sample size of IBD cases was observed among studies, ranging from ~100 to 20 000. The geographic distribution of sample sources of included gene-environment interaction studies is depicted in Figure 2. We grouped the studies into 3 categories: gene-smoking/lifestyle interaction (*N* = 26), gene-diet interaction (*N* = 15), and gene-microorganism interaction (*N* = 2), according to the environmental factors being studied (Figure 3).

Almost all included studies used a candidate gene approach focusing on a limited number of genes, except for 2 articles studying Immunochip-wide interaction and HLA region modification based on custom SNP arrays.^20,21^ Among the 39 eligible studies, there were 29 case-control studies, 4 cohort studies, 3 case-only studies, 2 cross-sectional studies, and 1 sib-pair linkage study; 23% of the included studies only conducted stratification analysis and 77% performed interaction testing. The characteristics of included studies is presented in Table 1.

**Table 1.** Basic information of included studies.

No.	First author	Year	Study design	Sample size (case/control)	Variants	Implicated gene	Environmental factors	Interaction analysis	Outcome	Main findings
1	Marie Pierik	2005	Sib-pair linkage study	892 sibling pairs	Microsatellites D14S261, D14S283, D14S972, and D14S275	*IBD4*	Smoking	Strata analysis Permutation test	CD risk	The significant linkage of IBD4 for CD was only observed in the families in which at least one of the affected siblings was an active smoker at the time of diagnosis.
2	Alexandra V. Pearce	2006	Case-control	1897 (1148/749)	R30Q (rs1248696)	*DLG5*	Smoking	Strata analysis Chi Squared test	CD risk	The frequency of *DLG5* R30Q is higher in CD cases who had ever smoked than those who never smoked. The frequency of A allele at the *DLG5* R30Q locus is 12.6% among the CD patients with smoking history, while for the nonsmokers, the frequency of A allele is 6.8%. *P*-values >.05 when comparing frequencies to controls.
3	Steven R. Brant	2007	Case-control	689 (353/336)	G908R, 1007fs, and R702W	*NOD2*	Smoking	Strata analysis Logistic regression model	CD risk	No significant effect modification of smoking on the association between *CARD15/NOD2* genotypes and CD risk was observed. No interaction effect size was presented.
4	Anja Ernst	2007	Case-control	1749 (953/796)	G908R, 1007fs, and R702W	*NOD2*	Smoking	Multiplicative interaction Logistic regression model	CD and UC risk	No significant multiplicative interaction effect of smoking and *NOD2* genotypes on the risk of either CD or UC was observed.
5	Elizabeth V. Fowler	2008	Case-control	3030 (1366/1664) *	*ATG16L1* T300A (rs2241880)	*ATG16L1*	Smoking	Additive interaction: Synergy index Multivariate logistic regression	CD risk	A positive additive interaction of smoking and *ATG16L1* T300A was observed regarding the risk of CD: Synergy index = 3.18 (1.29-7.85) The current smokers carrying GG genotype had seven times higher risk of CD than the nonsmokers carrying AA genotype at *ATG16L1* T300A. (*P *<.001, OR = 7.65, 95%CI, 4.21-13.91)
6	Frans van der Heide	2010	Case-control	1286 (310/976)	*IL23R*: rs11209026 and rs7517847 1q24: rs12035082 *ATG16L1*: rs2241880 5p13.1: rs9292777 and rs17234657 *IRGM*: rs13361189 and rs4958847 *IL12B*: rs6887695 IBD5: rs2522057 *DLG5*: rs2165047 *NKX2-3*: rs10883365 *CCNY*: rs3936503 10q21: rs10761659 *HERC2*: rs916977 *NOD2*: G908R, Cis1007fs, and R702W *PTPN2*: rs2542151	*IL23R* 1q24 *ATG16L1* 5p13.1 *IRGM* *IL12B* *IBD5* *DLG5* *NKX2-3* *CCNY* 10q21 *HERC2* *NOD2* *PTPN2*	Smoking	Strata analysis	CD risk	No interaction test was performed. The effect of *IL23R* (rs7517847), 5P13.1 (rs9292777 and rs17234657), *IRGM* (rs13361189 and rs4958847), *IL12B* (rs6887695), *DLG5*(rs2165047), *NKX2-3*(rs10883365), *CCNY* (rs3936503), *NOD2* (R702W) and *PTPN2* (rs2542151) on CD risk was modified by smoking status.
7	Vibeke Andersen	2010	Case-control	1613 (834/779)	*IL1B*: C-31T (rs1143627) *IL10*: C-592A (rs1800872), C-819T (rs1800871), and G-1082A (rs1800896) *HMOX1*: A-413T (rs2071746)	*IL1B* *IL10* *HMOX1*	Smoking	Multiplicative interaction Logistic regression model	CD and UC risk	Interaction test was conducted but interaction effect size was not reported. A significant interaction of *HMOX1* A-413T (rs2071746) and smoking regarding CD risk was found. (*P* interaction =.04) The interaction of *IL1B* C-31T (rs1143627) and smoking regarding UC risk showed a borderline significance. (*P* interaction =.05)
8	Anja Ernst	2010	Case-control	1749 (953/796)	GSTM1 null genotype GSTT1 null genotype GSTP1 Ala114Val and GSTP1 Ile105Val	*GST*	Smoking	Strata analysis Logistic regression model	CD and UC risk	No formal interaction measures were presented. The protective effect of *GSTM1* null genotype on UC risk was only observed in ever smokers but not in never smokers. (OR = 0.5, 95%CI not specified, *P* =.004) No interaction was found when combing the *GSTT*/*GSTM1* null genotypes and *GSTP1* genotypes.
9	Vibeke Andersen	2011	Case-control	2862 (1705/1157)	A-1195G (rs689466), G-765C (rs20417), T8473C (rs5275)	*COX-2*	Smoking	Strata analysis Logistic regression model	CD and UC risk	No significant interaction between any tested functional *COX2* genotypes and smoking was identified for either CD or UC risk (All *P* interaction >.05). Interaction effect sizes were not presented. The association between *COX-2* A-1195G and UC disease risk was observed mainly in the never smokers but not in the past or current smokers. Among never-smokers, *COX-2* A-1195G AG/GG carriers had higher risk of UC than the individuals with AA genotype. (OR = 1.47, 95%CI, 1.11-1.96, *P *=.01)
10	Katherine L. Helbig	2012	Case-only	1636*	R702W, G908R, and 1007fs	*NOD2*	Smoking	Multiplicative interaction Logistic regression model	CD risk	Interaction effect was quantified based on case only study design. A significant negative interaction of *NOD2* and ever smoking was found at 1007fs polymorphism. (OR = 0.53, 95%CI, 0.39-0.73, *P* interaction =7 × 10^-5^)
11	Ashwin N. Ananthakrishnan	2014	Case-control	1372 (1035/337)	rs3733829, rs1695, rs1800566, and rs2071746	*CYP2A6* *GSTP1* *NQO1* *HMOX1*	Smoking	Strata analysis Multiplicative interaction Multivariate logistic regression models	CD and UC risk	Interaction test was conducted but interaction effect size was not reported. Ever smokers showed an increased risk of CD compared with nonsmokers mainly among the individuals with AG/GG genotypes in *CYP2A6* but not among those with AA genotype. (*P* interaction =.001). Similarly, former and current smoking showed an increased risk of CD only in those with *CYP2A6* AG/GG genotype (*P* interaction =.0027). Former smokers had three times higher risk of UC than never smokers only in those with GG/AG genotypes at *GSTP1* rs1695 but not in those with AA genotype (*P* interaction =.012). No tested genetic variants in *NQO1* and *HMOX1* showed significant interactions with smoking in relation to CD or UC risk.
12	James D Doecke	2014	Case-control	1640 (993/1788) *	rs1004819, rs7517847, rs10489629, rs2201841, rs11209026, and rs1343151	*IL23R*	Smoking	Additive interaction	CD risk	Additive interaction between *IL23R* polymorphisms (rs1343151 and rs7517847) and smoking was observed but the interaction effect size was not presented.
13	Ming-Hsi Wang	2015	Case-control	9780 (2927/6853) *	rs1126510	*CALM3*	Smoking	Strata analysis Multiplicative interaction Logistic regression model	UC risk	The effect of *CALM3* (rs1126510) on UC disease risk was significantly observed in never smokers but not in smokers. (*P* interaction =.007) Interaction effect size was not reported.
14	Jun-hong Li	2016	Case-control	1012 (502/510)	rs744166	*STAT3*	Smoking	Strata analysis Generalized multifactor dimensionality reduction Logistic regression	CD risk	Interaction effect size of *STAT3* (rs744166) and smoking was not reported. Never smokers with TC/CC genotype had lower disease risk of CD than smokers with TT genotype at *STAT3* rs744166. (OR = 0.52, 95%CI, 0.31-0.82, *P* <.001)
15	M. Ellen Kuenzig	2017	Meta-analysis and Case-only	9064 from previous studies 627 from Alberta IBD consortium	R702W, G908R, and 1007fs	*NOD2*	Smoking	Multiplicative interaction Meta-analysis and logistic regression	CD risk	The negative *NOD2*-smoking interaction on CD risk was observed specifically at 1007fs. (OR = 0.74, 95%CI, 0.66-0.83, *P* interaction not reported) No significant *NOD2*-smoking interaction was detected at either R702W or G908R.
16	Pankaj Yadav	2017	Case-only	19 735	Immunochip dataset and imputed classical HLA alleles	Immunochip-wide SNPs	Smoking	Multiplicative interaction Logistic regression model	IBD risk	The multiplicative interactions of 64 SNPs and smoking on IBD disease risk were found. (*P* interaction <5 × 10^-5^)
17	B. M. Lang	2018	Case-control	1434	6 smoking quantity associated SNPs: *CHRNA5* (rs588765), *PPP1R3C* (rs1329650), *CHRNA3* (rs1051730), *CHRNB3*(rs6474412), *CYP2A6* (rs4105144) and *EGLN2* (rs3733829) Genetic risk score based on these 6 smoking associated SNPs (Smoking GRS) (Low genetic risk score (GRS) < 8, High GRS ≧ 8) A simplified GRS (rs588765 and rs1329650) 2 additional SNPs associated with ever-smoking and smoking cessation: *BDNF* (rs6265) and *DBH* (rs3025343)	*CHRNA5, PPP1R3C, CHRNA3, CHRNB3,* *CYP2A6,* and *EGLN2*	Smoking	Multiplicative interaction Logistic regression model Negative binomial regression model Cox proportional hazards model	Surgery frequency in CD Time to first surgery in CD Ileal involvement in CD Fistulising disease in CD Extent of disease in UC	1)*CHRNA5*(rs588765)*, CHRNA3*(rs1051730) and *PPP1R3C*(rs1329650) showed significant interactions with smoking for predicting the number of surgeries in CD patients. 2)Significant interaction effect of smoking GRS, consisting of 6 smoking quantity associated SNPs, and smoking at diagnosis on the number of surgeries in CD was found after correction of multiple testing. (OR = 1.22, Bonferroni-*P *=.001) 3)High smoking GRS accelerated the time to first surgery in CD patients who smoke. (HR = 2.27, 95% CI not reported, Bonferroni-*P* =.008) 4)The interaction of *BDNF* (rs6265) and smoking on ileal disease for CD patients was nominally significant (OR = 10.608, 95% CI not reported, *P* interaction =.042) 5)The interaction of *BDNF* (rs6265) and smoking on extensive disease in UC patients was nominally significant (OR = 0.233, 95%CI not reported, *P* interaction =.023) No significant interaction of any tested smoking related SNPs and smoking was observed for prediction of fistula in CD patients.
18	Kelly C. Cushing	2018	Case-control	608 (298/310)	Nicotine dependence associated variant: rs16969968	*CHRNA5*	Smoking	Multiplicative interaction Logistic regression model	IBD-related surgery	In UC patients, the interaction between *CHRNA5* (rs16969968) and smoking status was significantly associated with the risk of IBD-related surgery. (OR = 2.72, 80%CI. 1.42-5.32; *P* =.05) In CD patients, the interaction between *CHRNA5* (rs16969968) and current smoking regarding the risk of IBD-related surgery reached predefined significance level (OR = 2.88, 80%CI, 1.30-6.64, *P *=.1)
19	Ho-Su Lee	2019	Case-control	3973 (882/3091)	5413 variants at 29.5-33.5 Mb on chromosome 6 (HLA region)	*HLA*	Smoking	Strata analysis logistic regression model	UC risk	Interaction effect estimation was not performed. The association between rs6915986 in HLA region and HLA-DRB1*15:02 with the risk of UC was modified by smoking. *HLA*-DRB1*15:02 and rs6915986 were significantly associated with UC disease risk among never smokers but not in current or former smokers.
20	Lingying Dai	2021	Case-control	625 (268/357)	rs324015	*STAT6*	Smoking and alcohol use	Logistic regression model Cross-over analysis	UC risk	Interaction effect size was not reported. The joint effects of *STAT6* rs324015 genotype and smoking/alcohol use on UC disease risk were identified. The smokers with GG/AG genotypes at *STAT6* rs324015 had higher risk of UC than the nonsmokers with AA genotype. The alcohol consumers with AG genotype at *STAT6* rs324015 had increased risk of UC compared with nonalcohol consumers with AA genotype.
21	Alan Z.Yang	2022	Cohort study with both prospective and retrospective components	5661	Polygenic risk score	IBD associated genes	38 candidate environmental variables (Dietary pattern, geographic variables, drug and surgeries)	Multiplicative interaction Cox proportional hazards regressions with interaction terms	IBD risk	The elevated risk of UC conferred by previous smoking was attenuated in individuals with high IBD polygenic risk. (95% CI for hazard ratio of interaction term per standard deviation PRS: 0.79-0.98, adjusted *P* =.034) Previous oral contraceptive use decreased the risk of IBD and UC among the individuals with high polygenic risk but increased the risk of IBD and UC in those with lower polygenic risk. (95% CI for hazard ratio of interaction term per standard deviation PRS: 0.73-0.93 for IBD, adjusted *P* =.008; 0.71-0.96 for UC, adjusted *P* =.049) No significant interaction between individual SNPs and smoking or previous oral contraceptive use on the risk of CD or UC was observed.
22	Shuai Yuan	2022	Cohort study	2604	Polygenic risk score	IBD associated genes	Sleep duration and daytime napping	Multiplicative interaction model	CD and UC risk	No significant interaction was found between PRS and sleeping duration/daytime naps for either CD or UC. The association between sleep duration/daytime naps and IBD risk was only observed among the individuals at high genetic risk.
23	Yuhao Sun	2023	Cohort study	2283	Polygenic risk score	IBD associated genes	Healthy lifestyle score combining six lifestyle factors	Multiplicative interaction model	CD and UC risk	No significant interaction was found between genetic risk and the healthy risk score in either CD (*P* =.85) or UC (*P* =.87).
24	Linmin Hu	2024	Cohort study	2112	Polygenic risk score	IBD associated genes	Maternal smoking during pregnancy	Logistic regression model	CD and UC risk	No significant interaction between PRS and maternal smoking was found in either CD (*P* =.059) or UC (*P *=.598).
25	Catarina Sousa Guerreiro	2009	Retrospective case-control study	99 (36/63)	*IL6*-174 and *TNFα*-857	*IL6, TNF*	Dietary fat intake	Strata analysis Logistic regression model	CD disease activity Harvey and Bradshaw Index	No significant interaction was identified. (All interaction *P* >.05) High intake of saturated and monounsaturated fats as well as a high ω6/ ω3 PUFA ratio were associated with higher disease activity among individuals carrying the *TNF-α-*857 CT/TT genotype. High saturated fat intake and monounsaturated fat intake seemed to be associated with active disease in CD patients carrying *IL6-174* GC/CC genotype.
26	Paula Ferreira	2010	Retrospective Case-control study	99 (36/63)	*CASP9* + 93C/T *FASLG*-843C/T *PPARγ* 161C/T *PPARγ* Pro12Ala	Apoptotic genes: *CASP9, PPARG,* and *FASLG*	Dietary fat intake	Strata analysis Logistic regression model	CD disease activity Harvey and Bradshaw Index	No significant interaction was identified. (All interaction *P *>.05) The detrimental effect of high total fat intake and high trans-fat intake was more prominent in wild type carriers of the *CASP9* + 93C/T polymorphism than in the individuals with *CASP9* + 93C/T mutation. The deleterious effect of n-6 PUFA on CD disease activity was only significantly observed among the individuals homozygous for wild type of *FASLG*-843C/T.
27	Liljana Gentschew	2012	Case-control	798 (306/492)	29 SNPs in 7 genes: rs12095080, rs2294511, rs2294512, rs731828, rs10136454, rs12885300, rs225011, rs225012, rs225014, rs1800668, rs3763015, rs3792796, rs3792797, rs3828599, rs8177412, rs8177425, rs870407, rs10752294, rs11258337, rs17529609, rs7901303, rs11937742, rs13123725, rs1553153, rs17480524, rs2302565, rs7666342, rs1548357, rs5748469	*DIO1, DIO2, GPX1, GPX3, SEPHS1,* *SEPSECS,* and *TXNRD2*	Serum selenium concentration	Not specified	CD risk	The significant interactions between serum level of selenium and three SNPs in *SEPHS1* and *SEPSECS* were identified after adjustment of multiple testing. *SEPHS1* rs17529609(G): Estimate = −1.740, SE = 0.617, *P* interaction =.0048 *SEPHS1* rs7901303(T): Estimate = −1.117, SE = 0.420, *P* interaction =.0078 *SEPSECS* rs1553153(A): Estimate = −1.754, SE = 0.623, *P* interaction =.0048
28	Irina Costea	2014	Case-control	432 (182/250)	15 SNPs in 3 polyunsaturated fatty acids (PUFA) metabolic genes: rs1290617, rs1290620, rs2283612, rs8106799, rs11230815, rs17831757, rs968567, rs174579, rs174627, rs174577, rs174601, rs174602, rs498793, rs174547, rs174575	*CYP4F3, FADS1,* and *FADS2*	Dietary ω3, ω6 and dietary PUFA ω6/ ω3 Ratio	Strata analysis Logistic regression analysis	Pediatric CD risk	Interaction effect was tested but interaction effect size was not reported. The associations between the dietary PUFA ratio and risk of pediatric CD were dependent on *CYP4F3* (rs1290617 and rs1290620) and *FADS2* (rs11230815, rs17831757 and rs174627) genotypes. Interaction between rs11230815 in *FADS2* and dietary ω3 intake was observed regarding the risk of pediatric CD (*P* interaction =.042).
29	Shenglong Xia	2016	Case-control	312 (124/188)	*FokI* (rs2228570), *BsmI* (rs1544410), *ApaI* (rs7975232), and *TaqI* (rs731236)	*VDR*	Serum 25-hydroxy vitamin D	Logistic regression analysis Multiplicative interaction models	CD risk	Significant interaction between *VCR* genotypes and vitamin D deficiency was observed at *FokI*(rs2228570), *ApaI*(rs7975232) and *TaqI*(rs731236). Interaction of *FokI* (TC + CC) and vitamin D deficiency on the risk of CD: *P* interaction =.027, OR = 0.419, 95%CI, 0.19-0.91 Interaction of *ApaI* (CA + AA) and vitamin D deficiency on the risk of CD: *P* interaction =.024, OR = 0.309, 95%CI, 0.11-0.86 Interaction of *TaqI* (TC + CC) and vitamin D deficiency on the risk of CD: *P* interaction =.040, OR = 5.841, 95%CI, 1.08-31.54 Interaction of *BsmI* (GA + AA) and vitamin D deficiency on the risk of CD: *P* interaction =.800, OR = 0.618, 95% CI, 0.02-25.44
30	Hamed Khalili	2016	Nested Case-control study	1111 (371/740)	6 CD and UC risk variants involved in TH17 pathway from most recent GWAS meta-analysis: *JAK2* (rs10758669), *STAT3* (rs12942547), *CCR6* (rs1819333), *IL21* (rs7657746), *IL10* (rs3024505), and *IL23R* (rs11209026)	*JAK2, STAT3, CCR6, IL21, IL10,* and Genetic risk score	Dietary Potassium	Logistic regression model with multiplicative interaction terms	CD and UC risk	No significant interaction between dietary potassium and specific SNPs in *STAT3, JAK2, CCR6*, and *IL10* was observed regarding risk of UC or CD. (all *P* interaction >.15) The association of dietary potassium with risk of CD and UC seemed to be dependent on the genotype of *IL21* (rs7657746). (*P* interaction =.004 and *P* interaction =.01) Interaction effect size was not reported.
31	Hamed Khalili	2017	Nested case-control study	971 (321/650)	140 CD and UC associated SNPs from the most recent GWAS-meta-analysis	IBD susceptibility loci	Dietary total iron intake Dietary heme iron intake	Conditional logistic regression with a multiplicative interaction term	CD and UC risk	Significant interaction effect between *FCGR2A* (rs1801274) and dietary heme iron intake on UC risk was observed. (*P* interaction =7 × 10^-5^) Interaction effect size was not presented. No significant interactions between any of the CD or UC related susceptibility loci and total dietary iron intake on risk of CD or UC were observed.
32	Ashwin N. Ananthakrishnan	2017	Nested case-control study	735 (240/495)	*CYP4F3* (rs4646904, rs1290617, rs3794987, and rs2683037) *FADS1 (*rs174561 and rs174556) *FADS2* (rs3834458 and rs174575)	*CYP4F3, FADS1,* and *FADS2*	Dietary polyunsaturated fatty acids (PUFA)	Strata analysis Multiplicative interaction Conditional logistic regression models	CD and UC risk	The effect of ω3/ ω6 PUFA ratio on the risk of UC was modified by *CYP4F3* rs4646904 genotype. (*P* interaction =.049) High intake of total ω3 PUFA increased the risk of UC among the individuals with GT/TT genotype but not in those with the GG genotype at *CYP4F3* rs1290617. (*P* interaction =.02) Interaction effect size was not reported. No gene-diet interactions were observed in patients with CD.
33	Berkeley N. Limketkai	2020	Case-control	480 (240/240)	rs731236 and rs2282679	*VDR* and *DBP*	Serum 25(OH)D concentration	Conditional logistic regression	CD risk	No significant interactions between serum vitamin D level and genetic variants in *VDR* and *DBP* on the CD risk were identified.
34	Hui Zhao	2020	Case-control	823 (367/456)	rs6887695 and rs2288831	*IL12B*	Alcohol consumption Pack-years of smoking	Strata analysis Logistic regression model	UC risk	No interaction test was performed. The effect of *IL12B* (rs6887695) on the risk of UC was only observed in smokers and alcohol consumers but not in nonsmokers and nonalcohol consumers. Alcohol consumption modified the effect of *IL12B* (rs2288831) on the risk of UC.
35	Yoshihiro Miyake	2022	Case-control	1045 (384/661)	rs6887695	*IL12B*	Alcohol consumption Pack-years of smoking	Multiplicative interaction Logistic regression model	UC risk	The effect of history drinking on the risk of UC was modified by *IL12B* rs6887695 genotype. (*P* interaction =.0008) Interaction effect size was not reported.
36	Patricia L. Pringle	2015	Cross-sectional	846	IBD Genetic risk score (IBD-GRS) and *IL-23R*	Illumina Immunochip	Obesity-BMI	Logistic regression model with multiplicative interaction terms	CD complications: Penetrating disease Stricturing disease Perianal disease Bowel resection	No significant interaction effect of IBD risk genes/IBD-GRS and BMI on CD related disease complications was observed. (all *P* interaction >.28)
37	Kimberley W. J. Van Der Sloot	2016	Cross-sectional	482	IBD Genetic risk score (IBD-GRS)	Illumina Immunochip	Obesity-visceral adipose tissue (VAT) volume	Logistic regression model and log likelihood ratio test	CD complications: Penetrating disease Stricturing disease Perianal disease Bowel resection	No significant interaction effect of GRS and VAT volume on CD related disease complications was observed. (all *P* interaction >.12)
38	Natalia Elguezabal	2012	Case-control	466 (278/188)	rs4988235, rs2066844, rs2066845, and Cins1007fs	*LCT, NOD2*	Mycobacterium avium subsp. paratuberculosis (MAP)	Logistic regression model	CD and UC risk	No interaction between MAP infection and *LCT* or *NOD2* on risk of UC or CD was found. Effect size and *P* values for interaction terms were not reported.
39	Charles N. Bernstein	2007	Population-based case-control study	581 (287/294)	G908R and Cins1007fs	*NOD2*	Serum Mycobacterium paratuberculosis antibodies	Logistic regression model with interaction terms	CD and UC risk	No significant interaction between *NOD2* variants and M. paratuberculosis serology status was observed. However, there is a trend indicating that participants with both *NOD2* mutation and M. paratuberculosis seropositive are more likely to have CD. M. paratuberculosis seropositive was not significantly associated with risk of CD among *NOD2* non carriers (*P* value =.9, OR = 1.03, 95% CI, 0.64-1.65), while in *NOD2* carriers, M. paratuberculosis seropositive has a significant effect on the disease risk of CD (*P* value <.001, OR = 4.86, 95% CI, 2.45-9.65).

### 3.1.2. The quality assessment of included studies

STREGA grading resulted in 3 studies with high quality, 26 studies with moderate to high quality, and 10 studies with moderate to low quality. Lack of reporting potential bias and estimating prior sample size often limited study quality.

### 3.1.3. Gene-smoking interaction on the risk of IBD

The majority of investigated genetic variants are IBD susceptibility loci, with about 62.5% of the total number of studies focusing on gene-smoking interaction in IBD, followed by multiple metabolic enzymes and immune response regulation associated loci, smoking behaviour-associated loci, classical *HLA* loci, and Immunochip-wide loci.

### 3.2. IBD susceptibility loci

#### 3.2.1. NOD2-smoking interaction

The most widely investigated IBD susceptibility genetic variants for gene-smoking interactions are the three major *NOD2* coding variants (R702W, G908R, and 1007fs). Initially, first efforts were failed to detect significant differences in the distributions of *NOD2* variants based on smoking status due to small sample size.^22,23^ Until 2010, Van der Heide et al. firstly observed a divergent association of *NOD2* R702W and CD risk between smokers and nonsmokers through stratified allelic association analysis.^24^ No interaction analysis was performed due to the lack of sufficient smoking status in the control group.

A significant *NOD2*-smoking interaction effect was first identified in a case-only study by Helbig et al.^25^ The negative multiplicative interaction between *NOD2* and cigarette smoking, observed only at *NOD2* 1007fs polymorphism, suggested that the CD risk conferred by *NOD2* 1007fs was decreased by tobacco smoking.^25^ Subsequently, a meta-analysis with >9000 CD patients confirmed this negative interaction.^26^ An immunochip-wide study from IIBDGC replicated and complemented these findings with 2 additional negative interacting SNPs (rs2270368 and rs17221417) in the same region by conditional analysis.^20^

#### 3.2.2. ATG16L1-smoking interaction

A significant synergistic interaction effect on CD risk between *ATG16L1* T300A variant and smoking was reported in a multicenter population-based study with 1366 patients and 1664 controls, indicating that the joint effect of current smoking and *ATG16L1* T300A is larger than the sum of their individual effects.^27^ No external study was performed to replicate the results. Also, no significant effect modification of smoking or passive smoking in childhood was observed at the same *ATG16L1* locus in a Dutch IBD cohort by stratified analysis.^24^

#### 3.2.3. IL23R-smoking interaction

Multiple SNPs in *IL23R* demonstrated an association with CD susceptibility in Europeans.^3^ After stratifying by smoking status at diagnosis, a significant effect of *IL23R* rs7517847 on CD risk was found among nonsmokers but not among smokers.^24^ In addition, passive smoking in childhood was found to modify the association between rs11209026 in *IL23R* and CD risk in this study.^24^ No interaction was tested.

An Australian IBD study discovered similar results at rs7517847 and further quantified the joint effect of smoking and the common wild genotype at *IL23R* rs7517847 on CD risk.^28^ Moreover, the strongest combined effect of *IL23R* and current smoking was found at rs1343151.^28^ No replication studies have been performed to validate these results.

#### 3.2.4. IL12B-smoking interaction

A significant association between *IL12B* rs6887695 and CD susceptibility was observed only in nonsmokers.^24^ In contrast to UC, a significant association was only discovered among smokers in a Chinese Han population, while no significant interaction effect was observed in a Japanese study.^29,30^

#### 3.2.5. Other IBD-related loci

The remaining studies exploring IBD susceptibility loci and smoking interaction regarding IBD risk were shown in single studies and lack replication. The association between *CALM3* (rs1126510) and UC risk appeared to be modified by smoking.^31^ Furthermore, never smokers with *STAT3* TC/CC genotype at rs744166 showed the lowest relative risk of CD compared with current smokers carrying TT genotypes.^32^

The IBD4 locus harboring abundant immune regulation genes (eg, *IL25*, *IRF9*, *LTB4R*, etc.) demonstrated significant linkage with CD.^33^ Mean allele sharing of 4 microsatellites in IBD4 is more prominent in families with smoking siblings than in nonsmoking families.^34^ In addition, smoking showed effect modification on the association between 5p13.1 (rs17234657 and rs9292777), *NKX2-3* (rs10883365), *IRGM* (rs13361189 and rs4958847), *CCNY* (rs3936503) and *PTPN2* (rs2542151), and CD risk. In contrast, associations between 1q24 (rs12035082), *IBD5* (rs2522057), 1q21 (rs10761659), and *HERC2* (rs916977) with the risk of CD were homogenous between smokers and nonsmokers.^24^

### 3.3. Genetic polymorphisms of metabolic enzymes and immunomodulatory molecules

Cigarette smoke contains large amounts of toxic chemicals and free radicals, which induce an inflammatory response. Accordingly, apart from the IBD-associated loci, variants in diverse metabolism enzymes and inflammatory regulation-relevant coding genes were hypothesized to enhance or attenuate the impact of smoking on the susceptibility to IBD or vice versa.

#### 3.3.1. GST-smoking interaction

Glutathione S-transferases (GSTs) are detoxification enzymes catalyzing glutathione conjugation reaction with endogenous and exogenous compounds.^35^*GSTM1*, *GSTT1*, and *GSTP1* are the genes encoding common GSTs.

The *GSTM1*-null genotype, caused by homologous recombination of the left and right 4.2-kb repeats which results in the entire gene deletion and complete absence of GSTM1 enzyme activity, was significantly associated with decreased risk for UC in smokers alone.^36^ No interactions with smoking were detected when combining *GST* deletion genotypes and low activity genotypes.^36^ The PRISM study was unable to confirm this potential interaction as all patients were wild type for *GSTM1*.^37^

In the PRISM study, an interaction effect for *GSTP1* rs1695 and smoking was shown with the significant association of former smoking and increased UC risk only in individuals with AG/GG genotype but not in those with AA genotype (*P*_interaction_ =.012).^37^ For CD, this interaction was not significant after correction for multiple testing (*P*_interaction_ =.024).^37^

#### 3.3.2. HMOX1-smoking interaction

Heme oxygenase 1 (HO-1) is a stress-inducible isozyme of heme oxygenases demonstrating cytoprotective properties because of their capability to break down free haem and generate antioxidant.^38^*HMOX1* is the gene encoding for HO-1.^38^ In *HMOX1* promoter region, rs2071746 showed potential interaction with smoking on CD risk (*P*_interaction_ =.04).^39^ The joint effect of rs2071746 AT/TT genotype and current smoking on CD risk was quantified with an OR of 3.38 (2.15-5.33). In contrast, no interactions were observed between rs2071746 AT/TT genotype and smoking regarding UC risk (*P*_interaction_ =.49).^39^ The PRISM study did not replicate significant interactions using a different inheritance model.^37^

#### 3.3.3. COX-2-smoking interaction

Cyclooxygenase-2 (COX-2) is an inducible isoform of cyclooxygenases catalyzing the synthesis of prostaglandins. The interaction effect of 3 functional COX2 polymorphisms, A-1195G (rs689466), G-765C (rs20417), and T-8473C (rs5275), was not significant for either CD or UC risk.^40^

#### 3.3.4. NQO1-smoking interaction

NAD(P)H quinone oxidoreductase (NQO) catalyzes the reduction of quinones as well as a diverse range of different compounds. In the PRISM study, rs1800566 polymorphism in *NQO1* demonstrated effect modification on the association between smoking and CD susceptibility.^37^ Current smoking increased the risk of CD only among the individuals with AA/AG genotype but not in those with GG genotype.^37^ However, no significant interaction effect was observed with smoking regarding CD or UC risk (*P*_interaction_ =.08).^37^ No external studies have been performed to replicate these findings.

#### 3.3.5. Immunomodulatory molecule loci-smoking interaction

Interaction of *IL1B* (rs1143627) and former smoking on UC risk was of borderline significance (*P*_interaction_ =.05).^39^ The increased UC risk for former smoking was only seen in those with CT/CC genotype compared with never smokers with TT genotype.^39^ No interaction was seen for CD.^39^ In addition, 4 polymorphisms in *IL10* (rs1800872, rs1800871, 1800896, and rs3024505) did not demonstrate significant interactions with smoking on the risk of either CD or UC.^39^

The AG/GG genotype at *STAT6* rs324015 was associated with increased risk for UC only in those with a smoking history in a Chinese Han population.^41^ The combined effect of smoking and AG/GG genotype was estimated, but an interaction effect was not tested.^41^ No external studies have been performed to replicate these results.

### 3.4. Smoking behaviour-associated loci

Smoking behaviour-associated SNPs derived from large-scale GWAS were hypothesized to interact with smoking and exacerbate the disease progression of IBD. *CHRNA5* (rs588765), *CHRNA3* (rs1051730), and *PPP1R3C* (rs1329650) were reported to significantly interact with smoking for the prediction of number of surgeries in CD patients.^42^ A genetic risk score (GRS) composed of 6 smoking quantity-associated SNPs demonstrated a statistically significant interaction effect with smoking regarding the number of CD surgeries as well as the time to first surgery.^42^

In contrast, no significant interactions of smoking quantity GRS with smoking were observed for surgery in UC patients.^42^ However, *BDNF* (rs6265) significantly interacted with smoking for proctitis in UC patients.^42^ In addition, the interaction between *CHRNA5* (rs16969968) and smoking in UC (OR = 2.72, *P*_interaction_ =.05) seems more prominent than in CD for the risk of surgery within a differtent cohort, while no interaction effect was seen for time to surgery.^43^

*EGLN2* (rs3733829), 40-kb upstream from the 3’ end of *CYP2A6*, significantly interacted with smoking on CD risk (*P*_interaction_ =.0027), but not on risk of surgeries.^37^ However, the intergenic variant rs4105144 between *CYP2A6* and *CYP2A7* demonstrated no interaction effect with smoking for the risk of surgeries in either CD or UC.^42^

### 3.5. Custom SNP arrays

Interactions of Immunochip-wide SNPs and smoking regarding IBD risk were estimated in an IIBDGC case-only study.^20^ The interaction between 64 SNPs and smoking was associated with IBD risk (meta-analysis Wald test *P* <5.0 × 10^-5^, heterogeneity Cochrane Q test *P* >.05).^20^ Six unique classical HLA loci demonstrated multiplicative interaction effects with smoking on the risk of IBD (*P *<5.0 × 10^-5^).^20^

The effect modification of smoking on the association between classical HLA loci and UC risk was evaluated in a Korean case-control study through smoking-stratified analysis.^21^HLA-DRB1*15:02 was associated with UC risk only in never-smokers but not in current/former smokers.^21^ In contrast, the significant association between HLA-DQB1*06 and UC was only detected in current smokers (*P* =6.39 × 10^-12^).^21^ No interaction was tested. No significant multiplicative interaction effect of HLA-DRB1*15:02 or HLA-DQB1*06 with smoking was observed regarding either CD or UC risk in the IIBDGC case-only study at the significance level of *P *<5.0 × 10^-5^.^20^

#### 3.5.1. Gene and diet interaction on the risk of IBD

##### 3.5.1.1. Fat intake

The effect of dietary fat intake regarding CD and UC risk was highly heterogeneous across studies.^44,45^ Consequently, it was postulated that intake of dietary fat was associated with disease susceptibility or disease activity mainly among the individuals carrying certain variants in pro-inflammatory cytokine genes, apoptotic genes, or the genes involved in fatty acid metabolism.

No statistically significant interactions between high intake of dietary fats and *TNFα-*857 or *IL6*-174 genotypes were found in relation to CD disease activity.^46^ Nevertheless, a significant risk effect of high intake of saturated and monounsaturated fat as well as the ratio of ω6/ω3 polyunsaturated fatty acid (PUFA) on CD disease activity was observed mainly in subjects with *TNFα-*857 CT/TT genotype. Also, a trend toward an effect of high intake of saturated fat and monounsaturated fat on CD disease activity among *IL6-*174 GC/CC carriers was observed. No replication studies have been performed to reproduce these findings.

No significant interaction between fat intake and SNPs in apoptosis genes was detected in a follow-up study.^47^ However, high intake of total fat and trans-fat were associated with CD disease activity mainly in wild-type carriers with the *CASP9 + *93C/T polymorphism. On the contrary, the significant association between high monounsaturated fat intake and CD activity was mainly observed in the individuals with *CASP9 + *93C/T variant. In addition, the intake of saturated and monosaturated fat was associated with increased disease activity only in those with wild-type *PPARγ* Pro12Ala. What is more, high ω6 PUFA intake was more harmful in *FASLG-*843C/T wild-type carriers compared with those carrying *FASLG-*843C/T. No further studies have been conducted to replicate these results.

The interaction between 15 SNPs across 3 PUFA metabolic genes (*FADS1*, *FADS2*, and *CYP4F3*) and dietary ω6 PUFA, ω3 PUFA, and the ω6/ω3 PUFA ratio conferring susceptibility to CD has been explored in pediatric patients.^48^ No interaction between these 15 SNPs and ω6 intake was detected, but a significant interaction was observed between *FADS2* (rs11230815) and dietary ω3 intake in relation to pediatric CD risk (*P*_interaction_ =.042). Additionally, the association between dietary ω6/ω3 PUFA ratio and pediatric CD occurrence seemed to be significantly dependent on the *CYP4F3* genotypes (rs1290617 and rs1290620) and *FADS2* genotypes (rs11230815, rs17831757, rs968567, and rs174627).^48^

The Nurses’ Health Study (NHS) and NHS II did not replicate the interaction between rs1290617 in *CYP4F3* and PUFA ratio on CD risk (*P*_interaction_ =.94).^49^ However, in this study, significant interaction between higher intake of total ω3 PUFA and *CYP4F3* (rs1290617) regarding risk of UC was found (*P*_interaction_ =.02).^49^ In addition, the beneficial effect of high dietary ω3/ω6 PUFA ratio on risk of UC was only observed in the individuals carrying GG genotype at rs4646904 in *CYP4F3* (*P*_interaction_ =.049).^49^

##### 3.5.1.2. Alcohol

Two EAS studies have examined the interaction effect between polymorphisms in *IL12B* and alcohol use in relation to UC risk but yielded inconsistent results.^29,30^ The effect of *IL12B* polymorphisms on the risk of UC seemed to be modified by alcohol use in a Chinese population. rs6887695 CC/GC genotype and rs2288831 CC/TC genotype in *IL12B* were associated with increased risk of UC in alcohol consumers but not in those without a history of alcohol consumption.^30^ In contrast, ever alcohol consumption was associated with lower UC risk in individuals with CC genotype but not in those with GG/GC genotype at rs6887695 among a Japanese population (*P*_interaction_ =.0008).^29^

##### 3.5.1.3. Minerals: potassium, iron, selenium

High dietary intake of potassium before diagnosis demonstrated a protective effect on the risk of CD in NHS and NHSII.^50^ In addition, *IL21* (rs7657746) appeared to modify the association between potassium intake and CD susceptibility (*P*_interaction_ =.004).^50^ For individuals with the GG genotype at rs7657746 in *IL21*, high dietary potassium was associated with higher risk of CD (OR = 1.58 [1.15-2.16]) compared with lower consumption, while in those with AA genotype, high dietary potassium was associated with a reduction in risk of CD (OR = 0.90 [0.82-0.98]). Similarly, the beneficial effect of dietary potassium intake on risk of UC was only significantly detected in individuals with AA genotype at rs7657746 in *IL21* (*P*_interaction_ =.01).^50^ No significant interactions between dietary potassium and specific SNPs in *STAT3*, *JAK2*, *CCR6*, and *IL10* on CD or UC susceptibility were observed in this study (all *P*_interaction_ >.15). These results have not been replicated in independent studies.

The role of dietary iron and a possible interaction with established susceptibility loci on the risk of CD and UC were explored in NHS and NHSII.^51^ There was a significant interaction effect between *FCGR2A* (rs1801274) and dietary heme iron intake on UC risk (*P*_interaction_ =7.00 × 10^-5^).^51^ Increase of dietary heme intake appeared to be protective in relation to UC risk in women with the GG genotype but risk increasing among those with the AA genotype at rs1801274 in *FCGR2A*. No interaction between any CD-related susceptibility loci and dietary heme iron intake was found for CD patients.^51^ No replication studies have been conducted to reproduce these results.

Selenium deficiency frequently occurs in IBD and was found to be inversely associated with disease activity.^52,53^ Interactions between serum selenium and 29 SNPs in selenoprotein coding genes or genes controlling selenium synthesis were investigated in the cohort of 351 CD patients and 853 controls from New Zealand.^54^ Of these, 13 were found to significantly interact with selenium on the risk of CD. After adjustment for multiple testing, 3 significant interactions remained: rs17529609 (*P*_interaction_ =.0048), rs7901303 (*P*_interaction_ =.0078) in *SEPHS1*, and rs1553153 (*P*_interaction_ =.0048) in *SEPSECS*.^54^ These results have not been replicated.

##### 3.5.1.4. Serum 25-hydroxyvitamin D

No evidence was found of interactions between serum vitamin D and the Taq1 polymorphism in the vitamin D receptor (*VDR*) nor the polymorphisms in vitamin D binding protein on CD risk at multiple time points in a prospective cohort study of US military personnel.^55^ However, significant interactions between *VCR* polymorphisms (Fokl, Apal, and Taql) and vitamin D deficiency in relation to CD risk were observed in a Chinese case-control study.^56^ No interaction was found between the Bsml genotype in *VDR* and vitamin D deficiency on CD risk in this cohort.^56^

#### 3.6.1. Gene-microorganism interaction on the risk of IBD

Interactions between Mycobacterium para-tuberculosis (MAP) seropositivity and *NOD2* genotypes were not significantly associated with either CD or UC disease susceptibility.^57^ Nevertheless, *NOD2* mutation carriers with MAP seropositivity appeared more likely to have CD compared with those who were MAP seronegative and *NOD2* noncarriers.^57^ Mycobacterium para-tuberculosis infection did not significantly interact with variants in *NOD2* or lactase genes in relationship with CD or UC risk in a Spanish study.^58^

#### 3.6.2. Interaction between environmental factors and IBD polygenic risk

A UK biobank cohort study tested the multiplicative interactions of 38 candidate environmental variables and polygenic risk in predicting IBD occurrence.^59^ Significant protective interaction effect of polygenic risk score and previous smoking in relation to UC risk was discovered.^59^ In addition, previous oral contraceptive use demonstrated a divergent effect on IBD susceptibility among individuals with higher polygenic risk and lower polygenic risk, despite the fact that it had no direct significant association with IBD risk.^59^ No significant interactions between any perinatal/childhood exposures and polygenic risk were found in IBD, CD, or UC.^59,60^

Obesity, as a global epidemic problem, has been postulated to contribute to disease risk for IBD due to its pro-inflammatory effect.^61^ Effect modification of obesity on the association between CD GRS and disease complications has been assessed in 2 cross-sectional studies of CD patients enrolled in the PRISM cohort.^62,63^ No significant interaction effect was discovered in either study. Similarly, no significant interaction was found between polygenic risk and the healthy lifestyle score combining BMI, smoking, physical inactivity, drinking, sleep duration, and diet in either CD or UC.^64^ Sleep duration and daytime napping showed no interactions with polygenic risk regarding CD or UC risk.^65^

## 4. Discussion

To our knowledge, this is the first systematic review summarizing gene-environment interaction studies regarding IBD susceptibility, disease activity, and related complications. Of the 39 eligible publications, 22% (8/39) studies identified significant effect modification, and 47% (17/39) studies reported statistically significant interactions. Further meta-analysis was impossible due to incompletely reported interaction measures and the heterogeneity of study designs, genetic variants, and environmental factors.

In terms of gene-smoking interaction, *NOD2* coding variants were the most frequently studied. A negative interaction effect of *NOD2* Cis1007fs and smoking on the risk of CD was identified and replicated across different studies. The results of *NOD2* G908R and R702W were highly inconsistent. Apart from IBD risk loci, gene-smoking interactions were also observed for the polymorphisms in detoxification genes (*GSTP1* and *HMOX1*), inflammation modulators (*IL1B*), and smoking-associated genes; 64 SNPs were identified to interact with smoking by Immunochip-wide interaction study. But currently most gene-smoking interactions for IBD either lack replication or demonstrate inconsistent results across different studies.

Gene-diet interactions were reported for serum vitamin D with *VDR*, and for serum selenium with the selenoprotein genes. Interactions of dietary intake of fatty acids with PUFA metabolic genes were observed in pediatric CD and UC. The association of dietary intake of potassium in relation to the risk of CD and UC seems to be modified by rs7657746 in *IL21*. High dietary potassium demonstrated beneficial effects mainly among those with the AA genotype at rs7657746 in *IL21*. The risk of UC was significantly decreased for the ever-alcohol consumers compared with never-alcohol consumers mainly among the individuals carrying the CC genotype at rs6887695 in *IL12B*. High dietary heme iron intake reduced the risk of UC among women with the GG genotype but elevated the risk of UC among women with the AA genotype at rs1801274 in *FCG2RA*. Unfortunately, there have been no replication studies to confirm any of these findings. Similarly, there is very limited epidemiologic evidence of gene-microorganism interactions for IBD. Results of MAP infection and *NOD2* interaction that have been reported are contradictory.

One should be cautious in interpreting the current evidence of gene-environment interactions for IBD. For the purpose of prediction for disease outcomes in research, environmental factors and genetic variants both serve as markers, and introducing interaction terms may fit the data better and improve prediction accuracy. Similarly, if we are mainly interested in identifying high-risk population with no intention to disentangle the causal effects, assessing whether environmental exposures associate with disease risk differently based on genotypes can be sufficient. In this case, environmental exposures and genetic variants both work as proxies for the factors that are truly causally relevant for our outcomes. However, if disease etiology is the focus, reverse causality of environmental factors and potential confounders can undermine interpretation. For example, the longitudinal study of US military personnel cohort demonstrated that decreased serum 25-hydroxyvitamin D levels occurred after CD was diagnosed but not before, indicating that low vitamin D does not lead to the onset.^55^ Thereby, interaction of vitamin D deficiency and vitamin D-related genetic variants might not play a part in the development of IBD. Also, *NOD2* Cis1007fs was reported to negatively interact with smoking regarding CD risk in more than one study. However, the opposite prevalence of *NOD2* Cis1007fs and smoking across age at diagnosis was considered the main reason explaining this.^26^ Recent simulation analyses further showed that nonliner mediators of gene and environment, gene-environment correlation with nonlinearity, as well as genotype-stratified measurement errors in exposures can also cause spurious statistical interactions.^66^ Therefore, the confounders and potential mediators should be properly considered in the interaction analyses so that we can dissect the partition specifically contributed by biological interactions.

Prior sample size estimation was seldomly performed in the included studies. Most studies treated interaction analysis as “side dishes” without considering the sample size needed to properly power an interaction analysis. Therefore, nonsignificant interactions appeared frequently. In addition, the presentation of interaction results was often unsatisfactory. Presenting interaction effect sizes at multiplicative and additive scales with *P* values and confidence intervals is most informative for interpretation. It would also be recommendable to report the number of individuals exposed/unexposed to environmental factors, carrying/not carrying the risk variants, and with/without disease outcomes using 4 by 2 tables.^67^ This would allow researchers to recalculate and compare results across different studies and increase transparency.

Statistical interactions provided unfortunately far too limited information for biological interpretation. Despite that 64 smoking interacting SNPs were found by the largest gene-smoking interaction study in IBD, the underlying mechanisms are still poorly understood. In vitro and mice studies offered additional supportive evidence. For example, the risk of CD was 7-fold higher in the smokers with GG genotype at *ATG16L1*^T300A^ than nonsmokers with AA genotype. CD patients with *ATG16L1*^T300A^ genotype who are smokers had the most severe Paneth cell defects.^27^ Smoking-induced Paneth cell defects in *ATG16L1*^T300A^ mice were rescued by Peroxisome proliferator-activated receptor gamma (*Ppar*-γ) agonist rosiglitazone treatment.^68^ Thereby, in vitro and animal model studies can complement our knowledge of interactions between gene-environment regarding the IBD pathogenesis and lead us much closer to the potential therapeutic solutions.

The development of exposomics approaches such as high-resolution mass spectrometry empowers research to deeper characterize the comprehensive impact of chemical exposures on human health.^69^ Georg Braun et al. detected 473 chemicals in pregnant women plasma samples by quantitatively screening 1194 suspected target neurotoxicants,^70^ and 93 of those detected chemicals showed robust inhibition of neurite outgrowth.^70^ This fascinating study demonstrates the great power of biomonitoring in understanding the detrimental effects of chemical exposures for human health. Gastrointestinal tract is a main portal for the entries of massive external environmental substances in human body and serves as the first battlefield for the complex interactions across immune systems, microbiome, and toxic chemicals. Further applying systematic exposomics approaches to the studies of IBD can dramatically improve our understanding of the combinatorial effects of exposures regarding IBD risk.

GXE affects human health through multiple molecular mechanisms, such as gene expression, alternative splicing, chromatin state, and DNA methylation.^71^ Darina Czamara et al. found that the joint effect of genetic variants and prenatal environmental factors can best explain the DNA methylation in new-borns’ cord blood samples compared with individual effects.^72^ Although the altered molecular signatures of transcriptome and epigenome in IBD have been depicted regarding disease course and drug responses, there is not much evidence in the role of epigenome under the context of GXE in IBD.

The future of genome-wide SNPs-environment interaction has great potential to uncover the missing biological mechanisms of IBD. Evidence from the Immunochip-wide interaction study of gene and smoking for IBD has shown that most genetic modifiers of association between smoking and IBD risk are not IBD susceptibility loci.^20^ The Immunochip has a dense set of SNPs in MHC region but has a sparse coverage of the rest of the genome. So far, no genome-scale interaction study has been performed for IBD. Therefore, the missing heritability influenced by exposome factors is still poorly understood.

A large-scale GXE working group for IBD is needed to identify ancestry-specific gene-environment interaction effects. Currently, GXE working groups have been established for cardiovascular diseases, Type 2 Diabetes, and schizophrenia to facilitate investigation into the complex interplay of genetic variants and lifestyles.^73–75^ Standardized environmental measurement methods and genetic computational interaction models can be implemented in a well-designed infrastructure. GXE working groups enable future researchers to harmonize and compare the results from multiancestries, which would substantially increase the statistical power to determine the shared and ancestry-specific gene-environment interaction effects that could be used for preventive strategies in genetically susceptible individuals for IBD.

## Supplementary Material

jjaf061_suppl_Supplementary_Methods

jjaf061_suppl_Supplementary_Table

## Data Availability

The data underlying this article are available in the article and in its online supplementary material.

## Conference Presentation

Part of the results were presented at the European Crohn’s and Colitis Organisation Congress in 2024.
