# Supplementary material for: Gene-Environment Interactions in Inflammatory Bowel Disease: A Systematic Review of Human Epidemiologic Studies
Source: J Crohns Colitis. 2025 Jun 4;19(6):jjaf061. doi: 10.1093/ecco-jcc/jjaf061 (PMC12134891; doi:10.1093/ecco-jcc/jjaf061)
Supplement: jjaf061_suppl_Supplementary_Methods [file jjaf061_suppl_supplementary_methods.docx]

**Pubmed search strategy:**

("Inflammatory Bowel Diseases"[Majr] OR Inflammatory Bowel Disease*[tiab] OR ulcerative colitis[tiab] OR Crohn*[tiab])

AND

("Polymorphism, Single Nucleotide"[Mesh] OR "Genotype"[Mesh] OR "Genome-Wide Association Study"[Mesh] OR single nucleotide polymorphism*[tiab] OR genom*[tiab] OR polygenic risk score*[tiab] OR PRS [tiab] OR GWAS [tiab] OR GRS [tiab] OR genotyp*[tiab])

AND

("Environmental Exposure"[Mesh]OR "Environment"[Mesh] OR "Environmental Pollution"[Mesh] OR "Risk Factors"[Mesh] OR "Diet"[Mesh] OR "Diet, Western"[Mesh] OR "Smoking"[Mesh] OR "Breast Feeding"[Mesh] OR "Life Style"[Mesh] OR "Body Mass Index"[Mesh] OR "Vitamin D"[Mesh] OR "Gastroenteritis"[Mesh] OR "Human Migration"[Mesh] OR "Pets"[Mesh] OR "Income"[Mesh] OR "Educational Status"[Mesh] OR "Alcohol Drinking"[Mesh] OR "Diet, Food, and Nutrition"[Mesh] OR "Sports"[Mesh] OR "Exercise"[Mesh] OR "Floors and Floorcoverings"[Mesh] OR "Appendectomy"[Mesh] OR "Tonsillectomy"[Mesh] OR "Bronchial Hyperreactivity"[Mesh] OR "Hypersensitivity"[Mesh] OR "Lactose Intolerance"[Mesh] OR "Neuroticism"[Mesh] OR "Contraceptives, Oral"[Mesh] OR "Anti-Inflammatory Agents, Non-Steroidal"[Mesh] OR "Hormone Replacement Therapy"[Mesh] OR "Otitis Media"[Mesh] OR "Folic Acid"[Mesh] OR "Campylobacter"[Mesh] OR "Helicobacter hepaticus"[Mesh] OR "Mycobacterium avium subsp. paratuberculosis"[Mesh] OR "Salmonella"[Mesh] OR "Clostridioides difficile"[Mesh] OR "Microbiota"[Mesh] OR Diet*[tiab] OR body mass index[tiab] OR obes*[tiab] OR environment*[tiab] OR vitamin D[tiab] OR smok*[tiab] OR pollut*[tiab] OR Breastfe*[tiab] OR antibiotic*[tiab] OR Helicobacter pylori infection*[tiab] OR (childhood[tiab] AND hygiene[tiab]) OR Stress*[tiab] OR Sleep disturbance*[tiab] OR gastroenteritis[tiab] OR life style[tiab] OR Migra*[tiab] OR Pets[tiab] OR Income*[tiab] OR Educational level*[tiab] OR Educational Status*[tiab] OR Alcohol[tiab] OR Sport*[tiab] OR Physical activit*[tiab] OR Carpet*[tiab] OR Appendectom*[tiab] OR Tonsillectom*[tiab] OR Bronchial Hyperreactivity[tiab] OR Hypersensitivity[tiab] OR Lactose Intolerance[tiab] OR Neuroticism[tiab] OR bedpartner*[tiab] OR hot water[tiab] OR oral contraceptive*[tiab] OR Nonsteroidal Anti-Inflammatory Agent*[tiab] OR NSAID*[tiab] OR Hormone Replacement Therap*[tiab] OR Middle Ear Inflammation[tiab] OR Otitis Media[tiab] OR Folate[tiab] OR Campylobacter specie*[tiab] OR Helicobacter hepaticus[tiab] OR Salmonella specie*[tiab] OR Mycobacterium avium paratuberculosis[tiab] OR Clostridioides difficile[tiab] OR Microbiota*[tiab] OR Microbiome*[tiab])

AND

("Gene-Environment Interaction"[Mesh] OR G × E[tiab] OR interact*[tiab] OR Interplay[tiab])

NOT "Review" [Publication Type]

**Embase search strategy:**

('inflammatory bowel disease'/exp/mj OR 'ulcerative colitis'/exp/mj OR 'crohn disease'/exp/mj OR 'Inflammatory Bowel Disease*':ab,ti OR 'ulcerative colitis':ab,ti OR 'Crohn*':ab,ti)

AND

('single nucleotide polymorphism'/exp OR 'genotype'/exp OR 'genome-wide association study'/exp OR 'genetic predisposition'/exp OR 'single nucleotide polymorphism*':ab,ti OR 'genom*':ab,ti OR 'Polygenic risk score*':ab,ti OR 'GWAS':ab,ti OR 'PRS':ab,ti OR 'GRS':ab,ti OR 'genotyp*':ab,ti)

AND

('exposure'/exp OR 'environment'/exp OR 'pollution'/exp OR 'diet'/exp OR 'smoking'/exp OR 'lifestyle'/exp OR 'breast feeding'/exp OR 'body mass'/exp OR 'vitamin d'/exp OR 'gastroenteritis'/exp OR 'otitis media'/exp OR 'folic acid'/exp OR 'Campylobacter'/exp OR 'Helicobacter hepaticus'/exp OR 'Mycobacterium avium subsp. paratuberculosis'/exp OR 'Salmonella'/exp OR 'Clostridioides difficile'/exp OR 'microbiome'/exp OR 'microflora'/exp OR 'Diet*':ab,ti OR 'smok*':ab,ti OR 'pollut*':ab,ti OR 'body mass index':ab,ti OR 'obes*':ab,ti OR 'environment*':ab,ti OR 'vitamin D':ab,ti OR 'Breastfe*':ab,ti OR 'antibiotic*':ab,ti OR 'Helicobacter pylori infection*':ab,ti OR ('childhood':ab,ti AND 'hygiene':ab,ti) OR 'Stress*':ab,ti OR 'Sleep disturbance*':ab,ti OR 'gastroenteritis':ab,ti OR 'lifestyle':ab,ti OR 'otitis media':ab,ti OR 'folic acid':ab,ti OR 'campylobacter':ab,ti OR 'helicobacter hepaticus':ab,ti OR 'mycobacterium avium subsp. paratuberculosis':ab,ti OR 'salmonella':ab,ti OR 'clostridioides difficile':ab,ti OR 'microbiome*':ab,ti OR 'microbiota*':ab,ti)

AND

('genotype environment interaction'/exp OR 'Interact*':ab,ti OR 'Interplay':ab,ti OR 'G × E':ab,ti )

NOT

review:it

**Web of science search strategy:**

TS= ("Inflammatory Bowel Disease*" OR "ulcerative colitis" OR "Crohn*")

AND

TS= ("Genotyp*" OR "Genome Wide Association Stud*" OR "single nucleotide polymorphism*" OR "genom*" OR "polygenic risk score*" OR "PRS" OR "GWAS" OR "GRS")

AND

TS=("exposure*" OR "environment*" OR "Risk Factor*" OR "diet*" OR "Lifestyle*" OR "Exposome*" OR "body mass index" OR "obes*" OR "vitamin D" OR "smok*" OR "pollut*" OR "Breastfe*" OR "antibiotic*" OR "Helicobacter pylori infection*" OR ("childhood" AND "hygiene") OR "Stress*" OR "Sleep disturbance*" OR "gastroenteritis" OR "otitis media" OR "folic acid" OR " campylobacter" OR "helicobacter hepaticus" OR "mycobacterium avium paratuberculosis" OR "salmonella" OR "clostridioides difficile" OR "microbiome*" OR "microbiota*" OR "microflora*" )

AND

TS= ("Gene-environment interaction*" OR "genotype environment interaction*" OR "Gene × environment interaction*" OR "G × E" OR "Interact*" OR Interplay)

NOT Document Types: Review Articles

**Scopus search strategy:**

TITLE-ABS-KEY ("Inflammatory Bowel Disease*" OR "ulcerative colitis" OR "Crohn*")

AND

TITLE-ABS-KEY ("Genotyp*" OR "Genome-Wide Association Stud*" OR"Genetic Predisposition to Disease" OR "single nucleotide polymorphism*" OR "genom*" OR "polygenic risk score*" OR "PRS" OR "GWAS" OR "GRS" OR "SNP*")

AND

TITLE-ABS-KEY ( "exposure*" OR "environment*" OR "Risk Factor*" OR "diet*" OR "life style*" OR "Exposome*" OR diet* OR "body mass index" OR "obes*" OR "vitamin D" OR "smok*" OR "pollut*" OR "breastfe*" OR "antibiotic*" OR "Helicobacter pylori infection*" OR ("childhood" AND "hygiene") OR "stress*" OR "Sleep disturbance*" OR "gastroenteritis" OR "otitis media" OR "folic acid" OR " campylobacter" OR "helicobacter hepaticus" OR "mycobacterium avium paratuberculosis" OR "salmonella" OR "clostridioides difficile" OR "microbiome*" OR "microbiota*" OR "microflora*")

AND

TITLE-ABS-KEY ("Gene environment interaction*" OR {G × E} OR "interact*" OR "interplay")
