# Supplementary material for: Gene-Environment Interactions in Inflammatory Bowel Disease: A Systematic Review of Human Epidemiologic Studies
Source: J Crohns Colitis. 2025 Jun 4;19(6):jjaf061. doi: 10.1093/ecco-jcc/jjaf061 (PMC12134891; doi:10.1093/ecco-jcc/jjaf061)
Supplement: jjaf061_suppl_Supplementary_Table [file jjaf061_suppl_supplementary_table.docx]

| **Supplementary Table.** Excluded studies after full text screening | | |
| --- | --- | --- |
| No. | Reference title | Exclusion reason |
| 1 | Mushroom intolerance: a novel diet-gene interaction in Crohn's disease | Wrong outcome |
| 2 | Multi-omic analysis of the interaction between Clostridioides difficile Infection and pediatric Inflammatory Bowel Disease | No genotype data |
| 3 | The ATG16L1 risk allele associated with Crohn's disease results in a Rac1-dependent defect in dendritic cell migration that is corrected by thiopurines | Gene-gene interaction |
| 4 | Identifying environmental risk factors for inflammatory bowel diseases: a Mendelian randomization study | No interaction analysis |
| 5 | The effect of host genetics on the gut microbiome | Wrong outcome |
| 6 | Interplay of host genetics and gut microbiota underlying the onset and clinical presentation of inflammatory bowel disease | No interaction analysis |
| 7 | SMAD3 gene variant is a risk factor for recurrent surgery in patients with Crohn's disease | No interaction analysis |
| 8 | Inflammatory bowel diseases in Faroese-born Danish residents and their offspring: further evidence of the dominant role of environmental factors in IBD development | No genotype data |
| 9 | Host genetic and gut microbial signatures in familial Inflammatory bowel disease | No interaction analysis |
| 10 | Twin studies reveal specific imbalances in the mucosa-associated microbiota of patients with ileal Crohn's disease | No interaction analysis |
| 11 | The role of Vitamin D level and related single nucleotide polymorphisms in Crohn's disease | Wrong outcome |
| 12 | The ATG16L1–T300A allele impairs clearance of pathosymbionts in the inflamed ileal mucosa of Crohn’s disease patients | No environmental factors |
| 13 | Interactions between commensal fungi and the C-type lectin receptor Dectin-1 influence colitis | Animal model |
| 14 | T300A variant of autophagy ATG16L1 Gene is associated with decreased antigen sampling and processing by dendritic cells in pediatric Crohn’s disease | Wrong population |
| 15 | Genetic effects on the commensal microbiota in inflammatory bowel disease patients | Mediation analysis |
| 16 | Virus-plus-susceptibility gene interaction determines Crohn’s disease gene Atg16L1 phenotypes in intestine | Animal model |
| 17 | Protein tyrosine phosphatase non-receptor type 22 modulates colitis in a microbiota-dependent manner | Animal model |
| 18 | Anti-microbial antibody response is associated with future onset of Crohn’s disease independent of biomarkers of altered gut barrier function, subclinical inflammation, and genetic Risk | No Interaction analysis |
| 19 | Interaction between smoking and *ATG16L1T300A* triggers paneth cell defects in Crohn’s disease | No epidemiologic data |
| 20 | Bayesian Machine Learning Techniques for revealing complex interactions among genetic and clinical factors in association with extra-intestinal Manifestations in IBD patients | No interaction analysis |
| 21 | Inflammatory Bowel Diseases Before and After 1990 | No interaction analysis |
| 22 | Genetic liability for gastrointestinal inflammation disorders and association with gastrointestinal symptoms in children with and without autism | Wrong outcome |
| 23 | Crohn's Disease-Associated Pathogenic Mutation in the Manganese Transporter ZIP8 Shifts the Ileal and Rectal Mucosal Microbiota Implicating Aberrant Bile Acid Metabolism | Animal model |
| 24 | Transcriptomic analyses of treatment-naïve pediatric ulcerative colitis patients and exploration of underlying disease pathogenesis | Cell line |
| 25 | Evaluating the Effects of Omega-3 Polyunsaturated Fatty Acids on Inflammatory Bowel Disease via Circulating Metabolites: A Mediation Mendelian Randomization Study | No interaction analysis |
| 26 | IDENTIFYING ENTEROBACTERIACEAE VIRULENCE GENES ASSOCIATED WITH ACTIVE DISEASE IN ULCERATIVE COLITIS PATIENTS USING CULTURE-DEPENDENT AND -INDEPENDENT APPROACHES | Wrong publication type |
| 27 | Gene/environment interaction in the susceptibility of Crohn's disease patients to aluminum | In vitro study |
| 28 | Arabinoxylan and Pectin Metabolism in Crohn’s Disease Microbiota: An In Silico Study | No interaction analysis |
